# Planning for Serious Illness amongst Community-Dwelling Older Adults

**DOI:** 10.1155/2013/427917

**Published:** 2013-04-04

**Authors:** Donna Goodridge

**Affiliations:** College of Nursing, University of Saskatchewan, Room 421 Ellis Hall, 103 Hospital Drive, Saskatoon, SK, Canada S7N 0W8

## Abstract

Older adults have long been encouraged to maintain their autonomy by expressing their wishes for health care before they become too ill to meaningfully participate in decision making. This study explored the manner in which community-dwelling adults aged 55 and older plan for serious illness. An online survey was conducted within the province of Saskatchewan, Canada, with 283 adults ranging in age from 55 to 88 years. Planning for future medical care was important for the majority (78.4%) of respondents, although only 25.4% possessed a written advance care plan and 41.5% had designated a substitute decision maker. Sixty percent of respondents reported conversations about their treatment wishes; nearly half had discussed unacceptable states of health. Associations between key predictor variables and planning behaviors (discussions about treatment wishes or unacceptable states of health; designation of a substitute decision maker; preparation of a written advance care plan) were assessed using binary logistic regression. After controlling for all predictor variables, self-reported knowledge about advance care planning was the key variable significantly associated with all four planning behaviors. The efforts of nurses to educate older adults regarding the process of advance care planning can play an important role in enhancing autonomy.

## 1. Introduction

Given that almost three quarters of older adults lack decision-making capacity when urgent choices about life-sustaining treatment need to be made [1], older adults have long been encouraged by nurses and other health care providers to express their wishes for health care while they are healthy enough to meaningfully participate in treatment decision-making. Advance care planning has been recently promoted by the Centers for Disease Control and Prevention [2] as a process contributing to overall public health through its focus on supporting the individuals' health care choices and preventing unnecessary suffering. Widespread social marketing of advance care planning has made many excellent online and print resources available to the public [2–4].

Although advance care planning is now seen as an iterative process that includes the way in which people think about and communicate their values and preferences so that they may receive the health care they desire in the case of life-threatening illness [5], much of the extant research in this area has focused upon the completion of written advance care plans. Using a population-based approach, this study addressed the research question “How do community-dwelling adults aged 55 years and older plan for serious illness, either formally (e.g., by designating a substitute decision maker and preparing written advance care plans) or informally (e.g., by discussing states of health considered to be unacceptable to continue living or desired medical care in the event of serious illness)?” The overall objective of this study was to identify the associations between formal and informal planning for serious illness and key sociodemographic, health, and knowledge variables. Based on a review of current literature on advance care planning, it was hypothesized that sociodemographic variables (including age, gender, urban or rural location, education level, and income level), values (importance of planning for future care), and self-reported knowledge of advance care planning would be statistically significant predictors of behaviors relating to planning for serious illness. 

It is estimated that between 18% and 36% of all American adults in the general public have completed an advance directive [6]. Rates of advance directive completion amongst distinct subpopulations, such as persons with terminal illnesses and older adults, have been reported to range from 15% to 84.9% [1, 7–13]. Completion of an advance directive is associated with a wide range of factors, including “older age, greater disease burden, type and acuity of condition, White race, higher socioeconomic status, knowledge about advance directives or end-of-life treatment options, a positive attitude toward end-of-life discussions, a long-standing relationship with a primary care physician, and whether the patient's primary care physician has an advance directive” [6]. 

 While the clinical utility of advance directives has been rightfully questioned [14–20] and the uptake by intended consumers less than hoped in spite of widely available resources, support for the overall process of people considering and sharing key values and preferences for health care in the face of serious illness remains strong [5]. Social marketing campaigns have increasingly focused public attention on promoting dialogue, rather than completing forms, as a means of supporting the preferences and autonomy of individuals who may not be able to communicate his or her wishes for care in the future.

A number of studies have examined the extent to which discussions related to advance care planning are taking place, as well as the outcomes of these dialogues. Between 75% and 91% of participants over age 65 in the Canadian Study of Health and Aging [21] had considered who might make health decisions for them if they were unable to do this for themselves. The same study revealed that between 46% and 69% had discussed their preferences for end-of-life care with someone else. The benefits of having discussed preferences for future care were noted in a study of patients with advanced cancer by Wright and colleagues [22] and encompassed less aggressive medical care, including lower rates of ventilation (adjusted OR 0.26; 95% CI 0.08–0.83), resuscitation (adjusted OR 0.16 95% CI 0.03–0.80), ICU admission (adjusted OR 0.35; 95% CI 0.14–0.90), and earlier hospice admission (adjusted OR 3.37 95% CI 1.12–10.13), as well as better patient quality of life and improved bereavement adjustment. Discussions early in the trajectory of cancer have also recently been demonstrated to be associated with less aggressive treatment and greater use of hospice care [23].

Despite these positive outcomes, many people remain skeptical about the benefits of advance care planning, fuelled in part by society's denial of the inevitability of death [24, 25] and death's “sequestration” from mainstream society [26]. The choice *not* to engage in advance care planning is complex and highly individual but may include any one or more of the following reasons: believing that one's health is good and it is not necessary; believing that advance care planning is only for the terminally ill, elderly, or infirm; challenges discussing death; not being “ready”; lack of knowledge; difficulty completing the form; reluctance to broach the subject with the physician; fear of being a burden; incompatibility with cultural and spiritual traditions; preference to delegate treatment decision-making to family or others; lack of confidence that a written document would change the course of treatment received [15, 23, 27–30].

## 2. Materials and Methods 

### 2.1. Study Setting, Population, and Design

This study was conducted in April, 2012, within the province of Saskatchewan, a province located in Western Canada with a population just over one million people [31]. One third of the province's population is considered rural, with the remainder divided between two urban centres. 

The sample of 238 community dwelling older adults over the age 55 years represents a subgroup of the entire sample of 827 individuals obtained for a larger project, who were stratified by age, region, and sex to be representative of the population of the province. The entire sample was randomly selected from a pool of volunteers who had agreed to participate in online commercial market surveys (SaskWatch Research). Only data related to adults over age 55 years are presented in this analysis. This project received ethical approval from the University of Saskatchewan Behavioral Review Board. Participants provided consent for the data to be used in subsequent presentations and publications. 

### 2.2. Description of Variables

The survey was comprised of structured, closed-ended items salient to planning for serious illness care. Demographic data included age; sex; personal income; education; residence (urban or rural). Respondents were asked to identify the number and type of health conditions with which they lived from a list of common illnesses, as well as to respond to the following question: “How important do you think it is to plan for medical care at the end-of-life? (not at all important, somewhat important, important, or very important).” Respondents were asked to indicate their level of knowledge about advance care planning and living wills on a three-point scale (not at all familiar; some basic understanding; fairly or very good understanding). Those who reported a written living will or advance directive were also asked to identify sources of help received with preparing this document (consultation with lawyer; consultation with lawyer and family; consultation with family; consultation with someone else; prepared by myself).

### 2.3. Data Analysis

Statistical analysis was completed using SPSS 19.0. Given that an age of 65 years has been recognized as the point at which many Canadians retire from paid employment, respondents were divided into two groups: those 65 and younger and those older than 65 years. Level of significance (*σ*) was set at 0.05. Comparisons between the groups were completed using the Kruskal-Wallis test (with multiple Mann-Whitney tests adjusted with Bonferroni corrections for post hoc analysis) and chi-square tests of proportion where appropriate. In order to determine the characteristics influencing outcome behaviors variables (discussions of unacceptable states of health; discussion of wishes for treatment, designation of a substitute decision maker; preparation of a written directive), binary logistic regression was conducted. The strength of association was measured by the odds ratio (OR) and 95% confidence interval (CI). 

## 3. Results

Table 1 compares the demographic and health characteristics of the sample between respondents aged 55–64 years (younger group) and those who were 65 years and older (older group). Just over half (52.7%) of the 283 respondents were females. Respondents ranged in age from 55 to 88 years. The majority (82.1%) of all respondents had education beyond completion of high school. A higher proportion of the older group reported incomes of less than $60,000 than the younger group, while the opposite was true for incomes above $60,000. There were no differences between the age groups in location of residence. Participants residing outside of primary urban centres (populations >250,000) comprised 58.6% of the total sample. A significantly higher (*P* < 0.001) proportion of the older group reported four or more health conditions compared to the younger group, although the proportions were similar between the groups for those reporting one to three health conditions.

Table 2 compares the responses of the younger and older age groups in terms of values, knowledge, and behaviors related to planning for the end of life. The majority of respondents indicated they felt it was important or very important to plan for medical care in the event of serious illness (78.4%) and to plan for one's own funeral (62.2%). While 61.5% of respondents reported a good understanding of the term “living will,” substantially fewer indicated they had a good understanding of the term “advance care plan.” Approximately half of all respondents had discussed states of health in which they would find it unacceptable to live with someone close to them in the past year. One-third of those aged 65 and older reported they had a written directive compared to 20.5% of adults below age 65. Respondents in the older group were significantly more likely (*P* < 0.05) to have discussed wishes for treatment, designated a substitute decision maker, and to have prepared either an advance care plan or living will.

Respondents without a written advance care plan were asked to identify reasons for not preparing this document. The majority (60.0%) indicated they had not considered this yet, while 21.2% suggested that their families would make decisions about future health care for them and 18.2% indicated that their families together with their physician would decide. Only one respondent reported that care decisions would be made by the physician.

For the 72 respondents who had prepared a written advance care plan, the most common source of assistance to prepare the document was a lawyer (50%). A third of respondents cited families as a source of assistance, while 22.2% indicated they had completed the plan by themselves. Few (4.2%) respondents had the assistance of a health care professional in writing the directive.

Table 3 displays the adjusted association between the outcome variables (discussions about treatment wishes or unacceptable states of health; designation of a substitute decision maker; preparation of a written advance care plan) and the hypothesized predictor variables. After adjustment for all the variables listed in Table 3, women were twice as likely as men (O.R. = 2.05, 95% CI 1.16–3.62) to have discussed treatment wishes but were similar to men in terms of reporting discussion regarding unacceptable conditions, designating a substitute decision maker, or completing a written advance care plan. Those over 65 years of age were significantly more likely than those between the ages of 55 and 65 to have designated a substitute decision maker (O.R. = 2.30, 95% CI 1.32–3.99). The older group was not more likely to have completed a written advance care plan, although this association approached significance (*P* = 0.06). Education, income, and the number of self-reported health conditions were not associated with any of the four planning behaviors under consideration. Respondents from rural areas were significantly less likely (O.R. = 0.47, 95% CI 0.25–0.86) than those from urban centres to have completed a written advance care plan. Those who believed it was important to plan for care were more than twice as likely to have designated a substitute decision maker (O.R. = 2.32, 95% CI 1.15–4.70) and more than four times as likely to have completed a written advance care plan (O.R. = 4.31, 95% CI 1.42–13.07). Respondents who reported a good understanding of advance care planning had a significantly greater likelihood of engaging in all planning behaviors: discussion of treatment wishes (O.R. = 3.45, 95% CI 1.87–6.34); discussion of states of health they would find unacceptable to live with (O.R. = 5.62, 95% CI 2.92–10.92); designating a substitute decision maker (O.R. = 2.49, 95% CI 1.87–6.34); completing a written advance care plan (O.R. = 9.87, 95% CI 2.90–33.50). 

## 4. Discussion

The findings of this study demonstrate that neither formal nor informal planning for serious illness can yet be considered widespread amongst community-dwelling adults aged 55 and older. Formal planning for serious illness, in the form of designating substitute decision makers and preparing advance care plans, was reported by fewer than half of the respondents in this study. These proportions are similar to those reported in other studies [1, 7–13]. The proportion of respondents who engaged in informal planning for serious illness, such as conversations about treatment preferences and unacceptable health conditions, was only marginally higher, at 50% and 60%. 

In examining the demographic and health variables associated with planning behaviors, women were twice as likely as men to have discussed treatment wishes with someone close to them, but just as likely as men to have discussed unacceptable states of health, to have designated a substitute decision maker, or to have prepared a written advance care plan. Similar findings were reported by Garrett et al. [21], who reported women to be more likely to consider having a conversation about end-of-life wishes, but found no sex differences with respect to preparing an advance care plan.

In the adjusted model, there were no associations between age and informal planning behaviors. Older age proved significant only for the formal planning behavior of having designated a substitute decision maker, although the association between older age and having prepared an advance care plan approached significance (*P* = 0.06). Given the formal nature of both these activities and the fact that half of the advance care plans were completed with the assistance of lawyers, it may be that older adults are more willing to consider planning for serious illness under the umbrella of estate planning than health. Interestingly, the model found no associations between the number of health conditions reported by respondents and planning behaviors. It may be that an increasing number of health conditions are an expected sequela of the aging process and insufficient in and of themselves to trigger concern that planning for serious illness is warranted. 

The Transtheoretical Model [23] is increasingly used to explain variability in personal “readiness” to engage in advance care planning [20, 30]. The reason most frequently cited by respondents in this study for not preparing an advance care plan was that they “had not considered it yet.” Those individuals may have been in the “precontemplation” phase, described in the Transtheoretical Model, and were not ready to engage in formal planning behaviors. The decision to participate in planning for serious illness activities would, according to this model, mean that the “benefits” must outweigh the “costs” of the behavior perceived by the individual. The “costs” associated with the uncomfortable recognition and acceptance that one is personally vulnerable to poor health or death in the foreseeable future may have been such that respondents were not yet “ready” to engage in planning behaviors. If planning for serious illness is indeed a way to promote autonomy that is valued by older adults, it is worthwhile to further consider the factors that enhance readiness to participate in planning behaviors.

A significant proportion (21.6%) of respondents did not feel planning for future medical care to be important. Those who did not consider planning to be important were significantly less likely to have designated a substitute decision maker or have completed a written advance plan. While governments and health care providers continue to promulgate the benefits of planning for serious illness, it is important to bear in mind that a significant proportion of our patients may not share this value. Nurses who seek to educate patients about strategies to plan for future medical care must first take the time to appraise whether, in fact, a given individual believes these activities to be of value. Understanding the experiences that shape the individual's perspective on advance care planning is critical to a truly “patient-centred” approach.

Knowledge about advance care planning was consistently and independently associated with all types of planning for serious illness behaviors (discussions about treatment wishes or unacceptable states of health; designation of a substitute decision maker; preparation of a written advance care plan). The fact that respondents from rural areas, however, were only half as likely as those from urban areas to have an advance care plan suggests that access to information about planning for serious illness may, in fact, be a key driver for planning behavior. While the survey did not evaluate the access respondents had to education about advance care planning, access to health services is often limited in rural settings (CIHI). It is worthwhile to bear in mind, however, that the association between knowledge and behavior does not demonstrate causality, whereby more education about advance care planning leads directly to greater participation in planning. Individuals who were already receptive to the importance of planning for serious illness may have availed themselves of opportunities to educate themselves about this process. Nurses may play a significant role in identifying “teachable moments” and providing support and education to those who demonstrate an interest and willingness to learn more about ways to best plan for future serious illness. Tailoring interventions to the individual's level of readiness is referred to as “stage-matching” within the Transtheoretical Model [32].

## 5. Limitations

 While this survey was representative of the population of the province in terms of sex and region, the sample did not include the entire population of the province. Stratification by socioeconomic status or additional variables was not possible, although level of education may reflect socioeconomic status to some extent. Random selection occurred from the pool of those individuals who had agreed to participate in online surveys conducted by SaskWatch Research, and thus selection bias may have been present. Verification that ACP or LW documents were completed was not possible. 

## 6. Nursing Implications

 Supporting the autonomy of older adults is a key concern of nursing practice. Education of the patients with respect to the process of advance care planning has been recognized as a public health issue in which nurses may make a significant contribution. As advocates for supporting the autonomy of older adults, however, nurses who engage in practice related to advance care planning must keep in mind that, for almost one quarter of respondents in this study, planning for serious illness was not considered important. That some individuals do not hold planning as a value is an important consideration when planning nursing interventions designed to educate and foster participation in advance care planning, highlighting the need to match interventions to levels of readiness.

## Figures and Tables

**Table 1:** Demographic and health characteristics of the sample (by age group and overall).

	55–64 years (*N* = 171) %	≥65 years (*n* = 112) %	Overall (*N* = 283) %
Sex			
Male	45.6	47.3	47.3
Female	55.4	52.7	52.7
Education			
<or completed high school	20.1	14.4	17.9
Some post-secondary	52.1	55.0	53.2
Completed post-secondary	27.8	30.6	28.9
Annual personal income			
<$60,000	23.4^†^	42.0^†^	30.7
$60,000 or more	49.7^†^	28.6^†^	41.3
Refused	26.9	29.5	21.8
Residence			
Large Urban	43.3	41.1	41.4
Other	56.7	58.9	58.6
Number of health conditions			
None	27.5^†^	10.7^†^	20.8
1–3 conditions	58.5	55.4	57.2
4 or more conditions	14.0^*∧*^	33.9^*∧*^	21.9

^†^ *P* < 0.05 using the chi-square test for proportions with a Bonferonni correction.

^*∧*^ *P* < 0.001 using the chi-square test for proportions with a Bonferonni correction.

**Table 2:** Values, knowledge and behaviors related to planning for the end of Life.

	55–64 years (*N* = 171) %	≥65 years (*n* = 112) %	Overall (*N* = 283) %
Importance of planning for future medical Care			
Not at all or somewhat important	23.4	18.8	21.6
Important or very important	76.6	81.3	78.4
Importance of planning for own funeral			
Not at all or somewhat important	39.2	35.7	37.8
Important or very important	60.8	64.3	62.2
Familiarity with term “Living Will”			
Not at all familiar	7.0	6.3	6.7
Some basic understanding	32.7	30.4	31.8
Fairly or very good understanding	60.2	63.4	61.5
Familiarity with term “Advance Care Plan”			
Not at all familiar	28.1	23.2	26.1
Some basic understanding	35.1	37.5	36.0
Fairly or very good understanding	36.8	39.3	37.6
Discussed unacceptable states of health	53.2	43.8	49.5
Discussed wishes for treatment	53.8^†^	69.6^†^	60.1
Designated a substitute decision-maker	34.5^†^	52.7^†^	41.7
Prepared written LW or ACP	20.5^†^	33.0^†^	25.4

^†^ *P* < 0.05 using the chi-square test for proportions with a Bonferonni correction.

**Table 3:** Adjusted^1 ^associations between respondent characteristics and serious illness planning behaviors.

	Discussed treatment wishes	Discussed unacceptable conditions	Designated substituted decision-maker	LW or ACP completed
	OR (95% CI)	OR (95% CI)	OR (95% CI)	OR (95% CI)
Sex				
Female	2.05^‡^	1.25	1.21	1.47
(ref: male)	(1.16–3.62)	(0.72–2.18)	(0.70–2.09)	(0.77–2.80)
Age				
≥65 years	1.75	1.23	2.30^‡^	1.86
(ref: <65 years)	(0.98–3.11)	(0.71–2.13)	(1.32–3.99)	(0.99–3.50)
Education				
Some post-secondary education	1.26	1.59	0.93	0.98
(ref: ≤High school)	(0.60–2.65)	(0.76–3.29)	(0.45–1.93)	(0.41–2.33)
Completed post- secondary	1.64	1.72	0.70	0.79
(ref: ≤High school)	(0.7–3.81)	(0.75–3.92)	(0.30–1.60)	(0.29–2.13)
Annual personal income				
≥$60,000 (ref: <$60,000)	0.62 (0.31–1.22)	0.84 (0.43–1.61)	1.04 (0.54–2.00)	0.56 (0.26–1.21)
Unspecified (ref: <$60,000)	0.59 (0.29–1.26)	0.82 (0.41–1.62)	0.87 (0.44–1.71)	0.86 (0.40–1.85)
Rural residence (ref: urban)	0.83 (0.48–1.42)	0.86 (0.51–1.45)	0.65 (0.38–1.09)	0.47^†^ (0.25–0.86)
Health conditions				
1-2 conditions	1.14	0.81	1.06	1.05
(ref: 0 conditions)	(0.53–2.49)	(0.38–1.72)	(0.50–2.29)	(0.42–2.60)
3 or more conditions	1.20	1.30	0.96	0.85
(ref: 0 conditions)	(0.60–2.40)	(0.66–2.59)	(0.47–1.89)	(0.37–1.92)
Value				
Important to plan for care	1.67	1.24	2.32^†^	4.31*
(ref: not important)	(0.87–3.19)	(0.64–2.41)	(1.15–4.70)	(1.42–13.07)
Knowledge of ACP				
Understood term	3.45^*∧*^	5.62^*∧*^	2.49^‡^	9.87^*∧*^
(ref: not familiar)	(1.87–6.34)	(2.90–10.92)	(1.87–6.34)	(2.90–33.50)

^1^Adjusted for each of the variables listed in the table.

*P < 0.10.

^†^ *P* < 0.05.

^‡^ *P* < 0.01.

^*∧*^ *P* < 0.001.
